# Case report: 8 years after liver transplantation: *de novo* hepatocellular carcinoma 8 months after HCV clearance through IFN-free antiviral therapy

**DOI:** 10.1186/s12885-018-4175-2

**Published:** 2018-03-06

**Authors:** Giuliano Ramadori, Patrizia Bosio, Federico Moriconi, Ihtzaz A. Malik

**Affiliations:** 1 Department of Gastroenterology and Endocrinology, University Medical Center Goettingen, Robert-Koch-Street 40, D-37075 Goettingen, Germany; 2 General Practitioner, National health care system, Palazzago, BG Italy; 3 Gastro-Centro, Via Trevano 38, 6900 Lugano, Switzerland; 4 Institute of Anatomy and Cell Biology, University Medical Center, Kreuzbering 36, 37075 Goettingen, Germany

**Keywords:** Hepatocellular carcinoma, Liver transplantation, Hepatitis C virus, Interferon, Ribavirin

## Abstract

**Background:**

After orthotopic liver transplantation (OLT) for hepatocellular carcinoma (HCC), recurrent HCC mostly develops within 2 years. All cases of *de novo* HCC described so far occurred later than 2 years after OLT. Prevention of post-transplantation HCC has usually been tried to achieve by curing or controlling recurrent liver disease. This has been rationale for treatment with interferon (IFN)/ribavirin of HCV-recurrence in patients after OLT, transplanted for advanced HCV-induced liver disease and/or HCC. The availability of new and more efficient drugs has improved chances also for previously difficult-to-treat HCV-positive patients.

**Case presentation:**

A 75 year-old male patient who had undergone OLT for decompensated HCV-cirrhosis in 2009, and bilio-digestive surgery in 2011 under tracrolimus (0.5 mg/day) and prednisone (5 mg/day) immunosuppressive therapy, started to receive antiviral treatment for recurrent HCV-infection of graft with 200 mg/day ribavirin in combination with ledipasvir and sofosbuvir by the end of October 2015. Because of multiple side effects (anemia, asthenia, infections, and reduction of kidney functions - palliated by treatment with erythropoietin), treatment was stopped after 16 weeks. At the third control, a minimal increase in alpha-fetoprotein (AFP) serum level to 10 μg/L was measured 8 months after therapy, whereas both liver sonography and serum transaminases were normal. The patient’s general condition; however, remained poor, and a magnetic resonance imaging (MRI) of abdomen was performed 2 months later. A nodule of 3 cm in diameter with a pseudocapsule was found centrally in the liver. The patient had to be hospitalized for recurrent infections of the lung, overt ascites and peritonitis. Rapid tumor growth (10 cm) was detected during last stay in hospital (April 2017), concomitant with a rise of AFP-serum levels to 91 μg/L. The family decided to take the patient home, and best supportive care was provided by a general practitioner, local nurses and the patient’s dedicated wife until his death.

**Conclusion:**

Before treating OLT patients with HCV graft reinfection one should not only consider possible advantages of newly effective antiviral-therapies, but also life expectancy and possible side effects (difficult to manage at an outpatient service basis), including severe disadvantages such as the development of HCC.

## Background

Worldwide, approximately 180 million people are infected with the hepatitis C virus (HCV) [1]. Twenty to 30% of the infected people will develop liver cirrhosis, and among these, appr. 5% are in danger to develop liver cancer [2]. In the IFN era, about 50% of the patients infected with HCV genotype 1 (G1) achieved sustained virological response (SVR) [3]. HCV-infection is considered to be a major cause of liver cancer development [4], and infections may still develop in cirrhotic patients even after HCV-eradication [5]. Recently, an interesting French nationwide report pointed out that only 8% of the reported liver tumor cases were specifically due to HCV-infection [6]. This could be an explanation for the fact that, statistically, HCV-eradication may not guarantee longer life expectancy compared to persons not having received HCV-eradication [7]. HCV-induced liver cirrhosis and liver cancer are the key indicators for liver transplantation [8]. Chronic hepatitis and cirrhosis may quickly develop after reinfection of the graft in HCV-patients receiving orthotopic liver transplantation (OLT) for HCV-positive advanced liver cirrhosis or hepatocellular carcinoma (HCC) [9, 10]. In transplant centers, HCV-eradication therapy has been performed before and/or after transplantation, and successful viral elimination has been reported under combination of Peg-IFN and ribavirin, administered for different periods [11, 12]. Sustained virological response after both OLT and kidney transplantation has been shown to improve the overall survival in patients with HCV-infection [13–18].

Nowadays, anti HCV-therapies have been successively replaced by IFN-free direct-acting-antiviral (DAA) agents with or without ribavirin, leading to a reduction of therapy duration (12–24 weeks), and to an improvement of viral elimination rates to almost 100% for all difficulty grades and viral genotypes [19, 20]. In 2015, the combination of ladispovir / sofusbovir and ribavirin for 24 weeks in patients with HCV-reinfection after OLT was considered gold-standard, with therapy beginning at any time after transplantation [20].

Recurrence of HCC has been observed in a number of cases, even after successful elimination of HCV before or after OLT [21–24]. *De novo* HCC-development after OLT, however, is a very rare event, and only a few cases have been described so far [25]. A few reports demonstrated an increase of HCC development or recurrence in patients with liver cirrhosis due to HCV-infection and SVR after IFN-free treatment [26, 27] outside the transplantation setting. In contrast, *de novo* HCC development after having achieved SVR in patients transplanted for HCV-cirrhosis has not yet been described [25].

## Case presentation

**Fig. 1:**
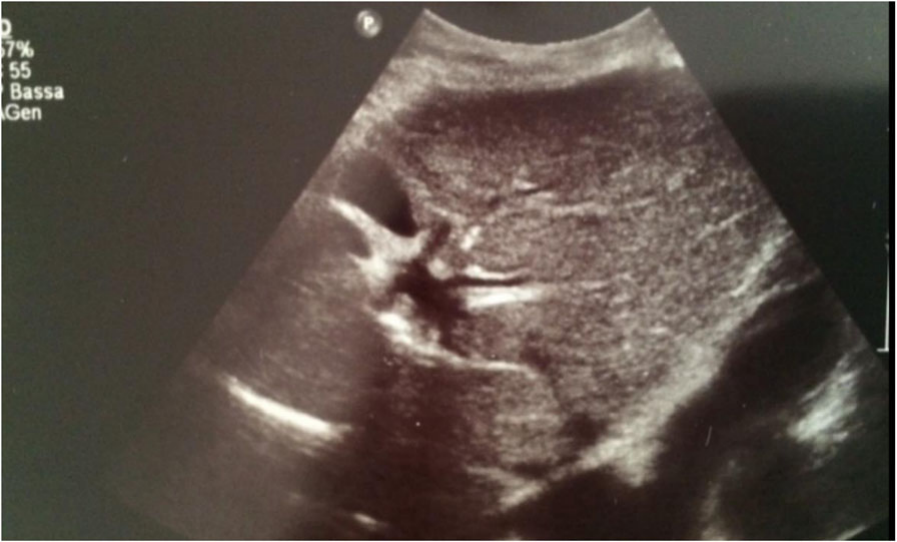
Liver ultrasound examination performed 10 months after the end of treatment when AFP was 10 ng/ml. No signs of liver cirrhosis and no signs of tumor were detectable

**Fig. 2:**
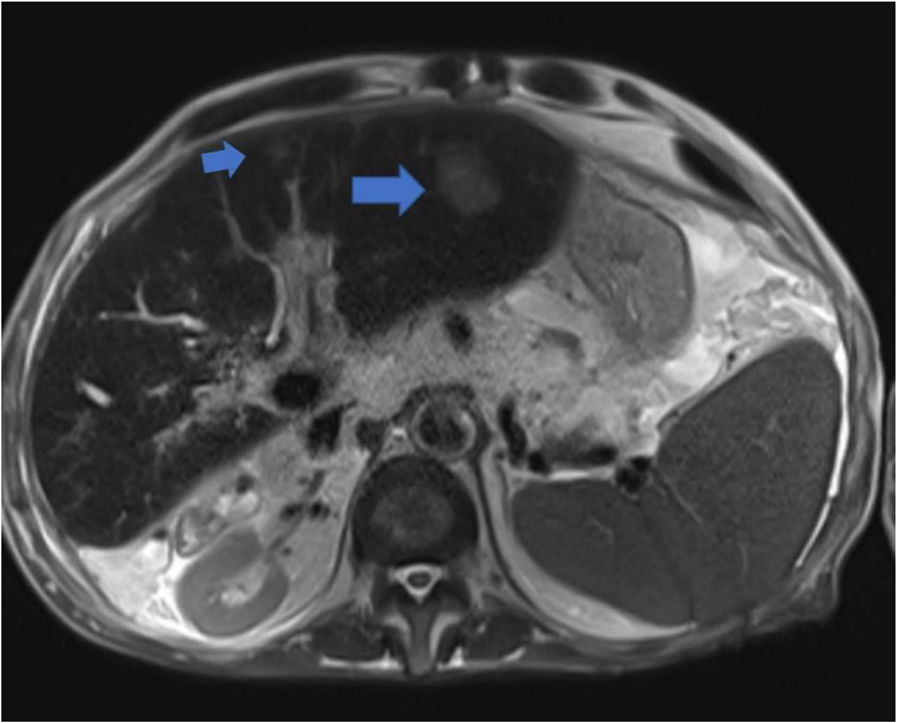
MRI-investigation of the liver 12 months after end of treatment with SVR; a nodule (3 cm diameter) with the characteristic of a HCC was detected in the left liver lobe (arrow), while a further small nodule was suspected (smaller arrow). No signs of cirrhosis were detectable

**Fig. 3:**
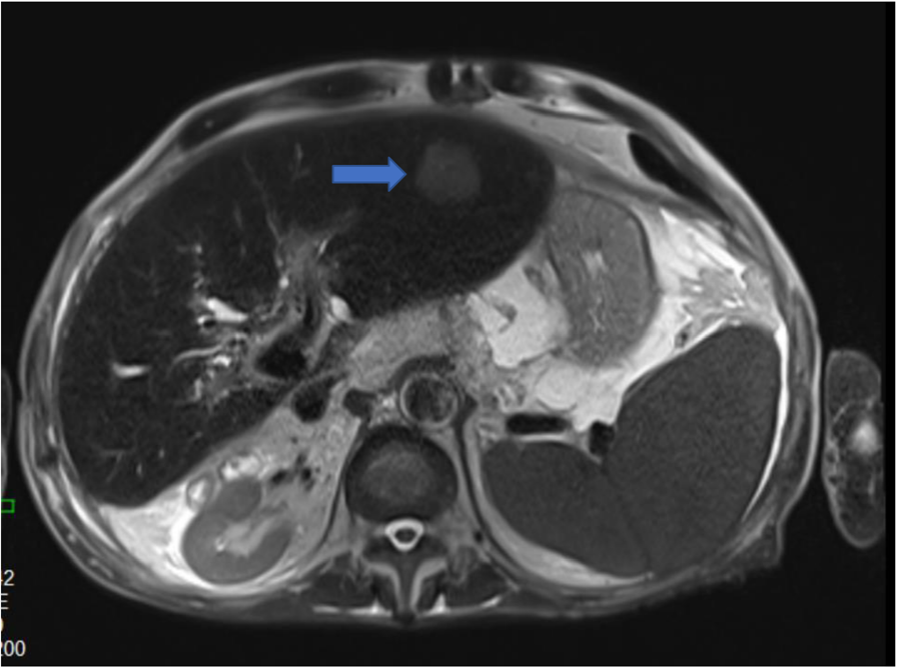
Additional slice of the MRI showing the nodule in S2 (arrow) 12 months after the end of treatment and SVR

**Fig. 4:**
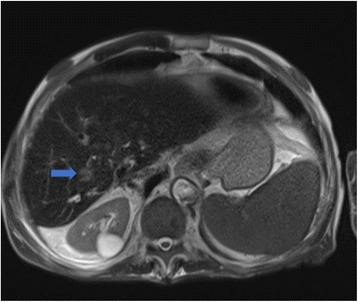
MRI investigation of the liver as described in Fig. 2. A third nodule (1.2 cm diameter) was described in the right liver lobe (arrow)

In July 2009, a 69-year-old male patient with decompensated HCV-cirrhosis underwent OLT. Postoperative time was complicated by bile duct anastomosis leakage, which was then treated with surgical bilio-digestive anastomosis in 2011. In 2012, liver histology showed bridging fibrosis, but no clear signs of cirrhosis. During the following years, a mild subclinical cholestasis was treated with oral deoxycholic acid. Immunosuppression was performed with tacrolimus (0.5 mg/day) and prednisone (5 mg/day). Despite viral reinfection and repeated episodes of cholangitis with pruritus, successfully treated with antibiotics, graft function was preserved with acceptable quality of life. As electrography on the graft reached 25 kPa, therapy with ledipasvir (90 mg) / sofusbovir (400 mg) combined with a reduced dosage of ribavirin (200 mg) was started by the end of October 2015. While HCV-negativity was found after 4 weeks of therapy, severe asthenia, loss of appetite and repeated infections developed together with progressive anemia. The latter persisted in spite of weekly subcutaneous administration of erythropoietin. Subsequently, reduction of kidney glomerular filtration rate developed. For this reason, therapy was stopped after 16 weeks while the patient developed ascites and edema of the legs in spite of improvement of liver stiffness down to 15 kPa. After two episodes of pneumonia and repeated laboratory and sonography surveillance (performed by experienced radiologists), 8 months after the end of treatment a first mild increase of serum AFP was detected, while ultrasonography (Fig. 1) did not detect any nodules in the liver. Two months later, a nodule of 3 cm size was detected by multiphasic-MRI (performed with administration of Gd-BOPTA) in the center of the left hepatic lobe (segment 2 (S2)), and a second smaller (1.5 cm) nodule in S4. The hyperintensity of the main lesion on T1-weighted images with enhancement in the arterial phase and with delayed portal venous wash-out [28] was judged to be suspicious for HCC according to the guidelines of the European Society for Medical Oncology (ESMO) (Figs. 2, 3 and 4). The multitude of the lesions led to the decision of therapeutic non-intervention, and best supportive care was offered to the patient in a tertiary care hospital.

**Fig. 5:**
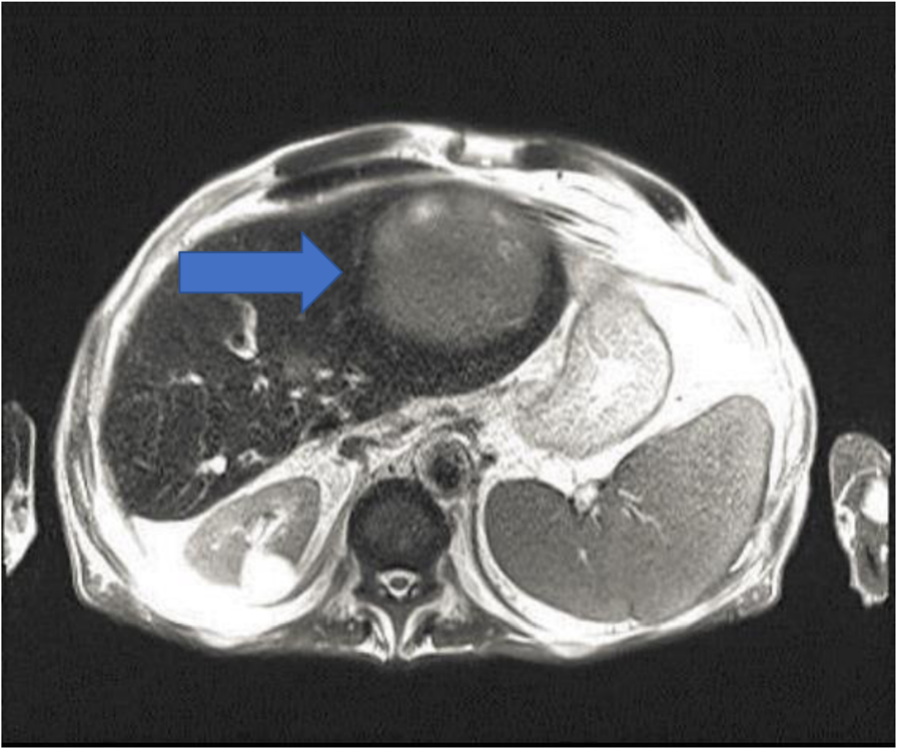
MRI-investigation 16 months after end of treatment. A rapid growth of the nodule in the left liver lobe was appreciable

**Fig. 6:**
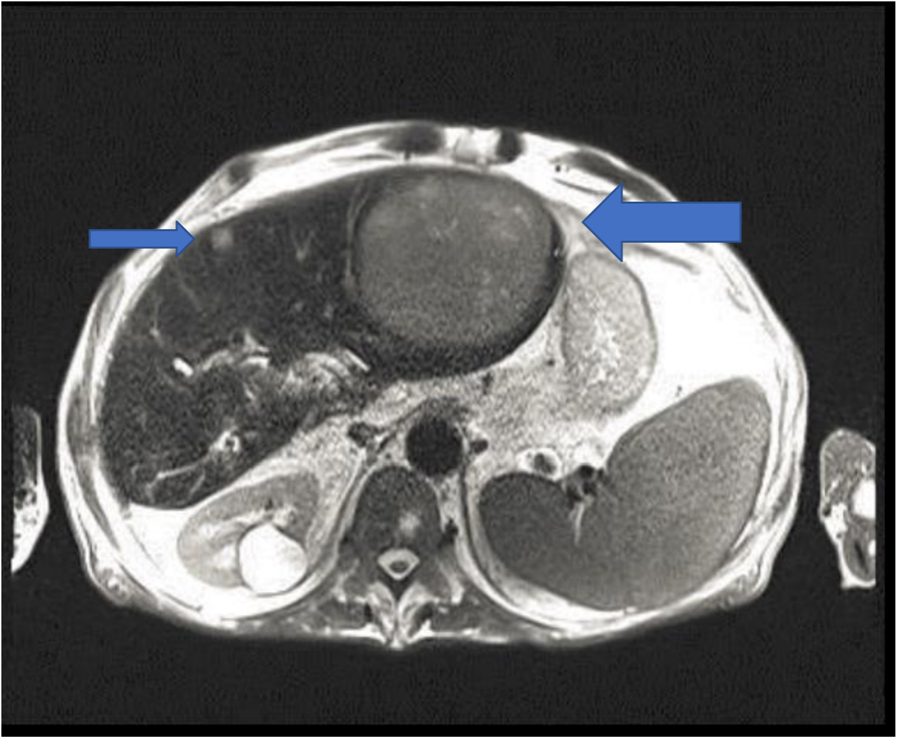
MRI of the liver as described in Fig. 2; the size of the subcapsular nodule (smaller arrow) has not increased compared to that of Fig. 1. The supposed third nodule (Fig. 4) was not confirmed

General physical conditions continuously worsened. An MRI of the liver performed by the end of April 2017 (Figs. 5 and 6) showed a massive increase in size of the former central nodule to 10 cm, completely occupying the left liver lobe, accompanied by an increase of serum AFP levels to 91 ng/ ml (Table 1). The family decided to take the patient home where he was having palliative care from his dedicated wife, from his home doctor and nurse.

**Table 1:** Characteristics of the patient with *de novo* tumor in the liver graft

	05/15	01/16	06/16	10/16	01/17	04/17
BW	63	63	65			68
ALT	55	25	15	12	17	11
AST	75	36	24	24	40	64
AP	295	304	269	326	285	320
YGT	291	295	210	287	267	315
BILI	1.01	1.28	1.03	0.92	0.86	1.35
TP	6.1	6.2	5.6	6.1	5.8	5.1
Alb^a^	3.8	3.8	3.6	3.7	2.9	2.8
Crea	1.1	1.08	1.36	1.7	1.26	1.86
PLT	138	137	129	203	140	182
INR	1.17	1.15			1.43	1.28
AFP		6.1	6.3	10.2		91
Fibro/Scan (kPa)	25	25	16	15.3		

^a^Albumin (20%) infusions were given depending on availability

## Discussion

HCV-infection alone or in combination with other damaging noxae such as alcoholic beverages or xenobiotics can lead to chronic hepatitis, cirrhosis and liver cancer (HCC). Risk of tumor development has been shown to be significantly reduced in patients who achieved sustained virological response (SVR) after antiviral therapy consisting of IFN-alpha and ribavirin. Long-term follow-up studies will reveal whether this is the case also for the highly effective new IFN-free therapies with directly-acting-antiviral (DAA) drugs with or without ribavirin [5, 29–32].

Decompensated liver cirrhosis and liver cancer still remain the principal indications for liver transplantation. Recurrence of HCV-infection in the graft, however, is a common adverse event immediately after liver transplantation. Reinfection and the concomitant administration of various drugs leads to the development of liver cirrhosis in about 20 to 30% of the transplanted patients within 5 years after transplantation, with reduction of overall survival compared to alcoholic liver disease [10]. The introduction of new drugs has ignited the discussion about the timing when therapy should be administered, namely before or after transplantation, and, if after transplantation, at which time point exactly?

Some reports have initiated a debate about the possibility of an increased frequency of liver cancer, which may be seen within months after the end of treatment in patients with SVR, who were previously operated for HCC-resection [26]. An increased incidence of HCC has also been reported in patients with liver cirrhosis after SVR achieved with IFN-free therapy [27]. This danger seems to be higher in older patients with more advanced cirrhosis [33–42]. Recurrence of liver tumor after OLT for HCC (mostly outside the liver) is a well-known complication, and the advantages of antiviral therapy during the patient’s stay on the waiting list [26, 43] or after OLT has not yet been proven.

Although we cannot exclude the possibility of the presence of tumor cells in liver at the beginning of DAA-therapy, this is the first case report on *de novo* liver cancer shortly after DAA-induced HCV-elimination in a patient who received long-term treatment after OLT. It is not yet clear how anti-viral treatment could be responsible for tumor development and/or acceleration of tumor growth. One possibility is that prolonged immunosuppression may be aggravated by antiviral agents (including ribavirin), thereby supporting the growth of scattered tumor cells, which have escaped immunosurveillance and were already present at the start of antiviral therapy.

Development of *de novo* HCC in patients transplanted for advanced liver cirrhosis without clinical signs of cancer is a very rare event. In fact, it has been thought that successful antiviral therapy and alcohol abstinence should prevent cirrhosis and carcinogenesis in the graft, and patients may rather die for other medical complications before the occurrence of cirrhosis and cancer.

Indeed, the early appearance of HCC in the graft after transplantation may represent the recurrence of a pre-existing tumor in the explanted liver, which was not detectable at the time of transplantation. This can only be proven through molecular analyses of the tumor material, which would allow the identification of donor or recipient origin. *De novo* HCC has also been reported in patients with HCV-reinfection, who achieved SVR after antiviral therapy with IFN and ribavirin [44]. Recurrence of cirrhosis has been confirmed to be a risk factor for *de novo* HCC-development in the re-infected graft, as is true for patients with HCV-positive liver diseases before transplantation. In one reported case, a tumor developed in a 59-year-old female patient transplanted for HCV-positive HCC, 6 years after living-donor liver transplantation and 2 years after signs of decompensated liver cirrhosis. She was successfully treated with antiviral therapy (Peg-IFN and ribavirin) for 48 weeks [44]. A CT-scan of the liver detected a hepatic nodule of 1 cm size, which was radiologically diagnosed as a classical HCC. The patient had higher AFP-serum levels during 6 months after CT-scan. Liver function tests were, however, within the normal range. Tumor resection was performed, and a tumor of 0.9 cm in size with trabecular growth and desmoplastic changes was diagnosed histologically. The non-tumorous liver tissue was cirrhotic with inflammatory infiltrates of the fibrotic septa. Genotyping of DNA samples extracted from the tumorous and non-tumorous tissue from the graft and the donor indicated that it was a *de novo* HCC, diagnosed 2 years after end of treatment, and about 2.5 years after confirmation of negative serum HCV-RNA level under antiviral treatment. Our patient developed detectable tumors and a minor increase in serum-AFP level 12 months after start of DAA-therapy and 8 months after the end of therapy. HCC was diagnosed based on the MRI-finding of liver tumor(s), and elevated AFP-serum level. However, in consideration of the reduced physical conditions and of the rather decreasing liver function parameters, in spite of normal transaminase serum levels, no further local or systemic anti-tumoral therapeutic measures were taken into consideration while a rapid and massive growth of the main tumor was detectable 4 months later.

At this time, patient had to be hospitalized for ascites edema of the legs, attacks of abdominal pain and fever. Recurrent bacterial peritonitis was treated with antibiotics. When diagnosis of a growing tumor was confirmed by MRI, he was sent home for best supportive care by his dedicated wife, home doctor and nurse until his death (June 12th, 2017).

## Conclusions

Decision of treating patients suffering of HCV-infection after liver transplantation with DAA-drugs should take in consideration possible side effects including cancer development and life expectancy.

## Abbreviations

AFP: Alpha-fetoprotein
DAA: Direct-acting antiviral
EPO: Erythropoetin
G1: Genotype 1
HCC: Hepatocellular carcinoma
HCV: Hepatitis C virus
IFN: Interferon
MRI: Magnetic resonance imaging
OLT: Orthotopic liver transplantation
SVR: Sustained virological response
